# The effects of self-esteem and peer relationship on the association of materialistic values with life satisfaction among Mexican-origin adolescents

**DOI:** 10.3389/fpsyg.2026.1944913

**Published:** 2026-10-14

**Authors:** Bing Shi, Zijun Ke, Richard Robins

**Affiliations:** 1 Sun Yat-sen University, Guangzhou, China; 2 University of California, Davis, Davis, CA, United States

**Keywords:** life satisfaction, materialistic values, Mexican-origin adolescents, peer relationship, self-esteem

## Abstract

**Introduction:**

Negative associations between adolescents' materialistic values (MV) and their wellbeing have been well documented; however, the underlying mechanisms driving this relationship remain insufficiently understood.

**Methods:**

Using longitudinal data from 674 Mexican-origin adolescents, this study addressed this gap with adoption of structural equation modeling (SEM) approach.

**Results:**

A negative association between materialistic values and life satisfaction (ages 14 & 16) was found. Furthermore, the findings revealed that higher materialistic values predicted lower self-esteem, which in turn led to decreased life satisfaction. Same-sex—but not opposite-sex—peer relationship served as an alternative explanatory factor. This study demonstrated self-esteem, as compared to same-sex peer relationship, played a more substantial role in mediating the relation between materialistic values and life satisfaction. Gender similarity—rather than gender difference—was identified in these processes.

**Discussion:**

These findings should have significant implications for educators seeking to design intervention programs aimed at mitigating adverse effects of materialism on adolescents.

## Introduction

As the primary socialization agent shifts from parents to peers in adolescence, building peer connections and forming a social identity among peers becomes crucial (Erikson, 1994; Mishra et al., 2026). This task often leads adolescents to engage in shopping and consumption (Benish-Weisman, 2024; John, 1999). Through these activities, adolescents develop materialistic values (MV), values which reflect the importance of material possessions, and the degree to which acquisition of the possessions is perceived as essential or desirable for achieving end states such as happiness and popularity among peers (Richins and Dawson, 1992; Shi et al., 2024).

Materialistic individuals view possessions as essential for achieving happiness and popularity (Richins and Dawson, 1992; Shi et al., 2024). However, self-determination theory (SDT; Grassinger et al., 2024; Ryan and Deci, 2000) posits that such values are unlikely to enhance life satisfaction—an important component of subjective wellbeing (Buecker et al., 2023). Two forms of goal pursuit—extrinsic and intrinsic—are proposed in SDT (Grassinger et al., 2024; Ryan and Deci, 2000). Correlational and experimental studies suggest that intrinsic goal pursuit (e.g., pursuit of close relationships, seeking autonomy, building up competence and physical health) promotes subjective wellbeing, whereas extrinsic goal pursuit (e.g., pursuit of social recognition, material wealth, and beauty) does not (Bradshaw et al., 2023; Kasser and Ahuvia, 2002; Roberts, 2026). Furthermore, materialistic individuals (vs. less-materialistic) engage more in extrinsic goal pursuit (Kasser, 2016; Otero-López et al., 2023), less likely to invest on intrinsic goal pursuit which satisfies the basic psychological needs of competence, autonomy, and relatedness, thereby is more beneficial for subjective wellbeing than the pursuit of extrinsic goals (Bradshaw et al., 2023; Lo et al., 2025; Ryan et al., 2008). Empirical research reliably indicates the desire for wealth and possessions (relevant to extrinsic goal pursuit) detriments subjective wellbeing across cultures and political contexts (e.g., Barnfield, 2020; Bradshaw et al., 2023; Moldes et al., 2025).

However, inadequate research has hitherto examined the mechanisms underlying the negative association of MV with life satisfaction. We aim to address this gap. Moreover, Mexican-origin adolescents are recruited for investigation, as Latinxs represent one of the largest and fastest-growing ethnic groups in the United States and continue to experience lower socioeconomic status as compared to other ethnic groups (Barger et al., 2009; Errisuriz et al., 2024). Based on the identified negative relation between MV and subjective wellbeing, higher MV would also relate with lower life satisfaction among Mexican-origin adolescents.

Based on the above-discussed SDT (Grassinger et al., 2024; Ryan and Deci, 2000), the endorsement of MV often leads an individual to concentrate on extrinsic goal pursuit (Kasser, 2016; Otero-López et al., 2023), while investing less on intrinsic goal pursuit. Note that, intrinsic, but not extrinsic, goal pursuit likely promotes life satisfaction through satisfying the basic psychological needs of autonomy, competence, and relatedness (Bradshaw et al., 2023; Lo et al., 2025; Ryan et al., 2008). The association of higher MV with lower life satisfaction would potentially occur through reduced satisfaction of the three psychological needs (i.e., needs of autonomy, competence, and relatedness).

Moreover, reduced satisfaction of psychological needs of autonomy and competence induces more negative self-concepts (e.g., Heppner et al., 2008; Lindgren et al., 2025; Valero-Valenzuela et al., 2021). Correspondingly, a substantial body of evidence in the MV literature indicates that adults with higher (vs. lower) MV score lower on self-concept certainty and clarity (Chang et al., 2024; Noguti and Bokeyar, 2014). Individuals who prioritize MV over other values express more self-doubt (Chang and Arkin, 2002; Dittmar and Isham, 2022) and display lower self-esteem (Ruvio et al., 2014; Zheng et al., 2024). Materialistic individuals, compared to less materialistic counterparts, exhibit greater financial anxiety (Ahamed and Limbu, 2024; Sinha et al., 2021), consume more impulsively (Pellegrino et al., 2022) and ultimately incur more debt (Kasser, 2016; Mabina and Mukwevho, 2024). Consequently, endorsement of materialistic values would escalate their insecure or uncertain self-perceptions. Taken together, we hypothesize higher MV would significantly predict lower self-esteem among Mexican-origin adolescents (H1).

In the literature, higher self-esteem is found to significantly correlate to higher levels of perceived life satisfaction (e.g., Kurnaz et al., 2020; Yang et al., 2024). Findings in previous research (Kurnaz, 2023; Kurnaz et al., 2020) indicate a positive, moderately significant effect between life satisfaction and self-esteem. Thus, we predict self-esteem would mediate the negative association of MV with life satisfaction: Higher MV would significantly predict lower self-esteem, which would lead to lower life satisfaction among Mexican-origin adolescents (H2).

Regarding the psychological need of relatedness, it affects one's engagement with interpersonal relationships. Based on SDT (Grassinger et al., 2024; Ryan and Deci, 2000), the endorsement of MV would decrease one's relatedness satisfaction. Materialistic values, or beliefs that link wealth and consumption to success and happiness, negatively influence interpersonal relationships (e.g., Manchiraju et al., 2021; Moldes, 2025). Materialism correlates to loneliness (Loh et al., 2021; Wang et al., 2025). Three studies (Moldes, 2025) employing correlational and experimental methodologies also show that materialism leads to heightened expectations and standards for a significant other, which are associated with poorer interpersonal outcomes.

Note that peer relationship involves same- and opposite-sex peers in adolescence. Although early adolescents exhibit gender-segregated peer interaction patterns (Leaper et al., 2022; Xie and Shi, 2009), over time, with increasingly puberty-related changes, both physical and psychological (Koenig et al., 2025), youth interact with opposite-sex peers more intensely. Given the identified negative association between MV and peer relationship, and the importance of same- and opposite-sex peers to adolescents (Gonzalez Avilés et al., 2021; Koenig et al., 2025; Syafiyah et al., 2025), we hypothesize higher MV would significantly predict lower same-sex peer relationship (H3) and lower opposite-sex peer relationship (H4) among Mexican-origin adolescents.

Satisfaction of the need of relatedness to important others (i.e., peers) is critically influential to adolescents' subjective wellbeing (Allen et al., 2021; Hall et al., 2023). A voluminous literature does indeed support a strong association of social relationships with happiness and wellbeing (e.g., Alsarrani et al., 2022; Hall et al., 2023). In a review by Alsarrani et al. (2022), a positive association between peer connection and life satisfaction has been reported and poor peer relationship is associated with loneliness in the majority of the extant studies. We thus hypothesize higher MV would significantly predict lower same-sex peer relationship, which would lead to lower life satisfaction among Mexican-origin adolescents (H5). Similarly, opposite-sex peer relationship would play this mediating role (H6).

The impact of gender on the mediation processes is furthermore examined. According to the social role approach, males are characterized as agentic and females as communal (Block et al., 2025; Stein et al., 1992). Studies based on a sample of school adolescents (e.g., Flouri, 2004; Shi et al., 2024) indicate that boys are more likely than girls to endorse materialistic values. A number of studies demonstrate higher materialism scores for men than for women (e.g., Saleem and Tariq, 2025; Segal and Podoshen, 2013). It appears that being materialistic would be less socially acceptable among girls than among boys. In addition, previous research suggests that the association of higher materialism with more insecure or uncertain self-perceptions is stronger for females than for males (Noguti and Bokeyar, 2014; Saleem and Tariq, 2025). The negative relationship between materialistic values and self-esteem would be more pronounced among girls than among boys. In addition, Luijten et al. (2023) have reported that the longitudinal effect of global self-esteem on wellbeing is significant for girls but not for boys. Overall, we predict the mediating effect of self-esteem, as hypothesized in H2, would be stronger among girls than among boys (H7).

Moreover, the negative association of MV with peer relationship would be stronger among girls than among boys considering the lower levels of social acceptance of being materialistic among girls than among boys (Block et al., 2025; Felix et al., 2013; Saleem and Tariq, 2025). Regarding gender differences in the association of peer relationship with life satisfaction, findings from quite few empirical studies in the extant literature are mixed: On the one hand, as girls are socialized to value relationship more (Block et al., 2025; Rose and Rudolph, 2006) and more sensitive to social influence (Rudolph and Conley, 2005; Orben et al., 2022), the link between peer relationship and life satisfaction would be stronger among girls than among boys; In contrast, Luijten et al. (2023) have reported a strong relation between close friendship and subjective wellbeing among boys, but not among girls. Given the likelihood of a stronger negative association between MV and peer relationship among girls (vs. boys) and the mixed findings of gender differences in the association between peer relationship and life satisfaction, we examine gender differences in the mediation processes of peer relationship (H5-6) without directional hypotheses.

In summary, we aim to investigate the underlying mechanisms for the negative association of MV with life satisfaction among Mexican-origin adolescents. With adoption of SEM approach, Hypotheses 1–7 were examined (as summarized in Table 1). Findings should have critical contributions to understanding the mechanisms accounting for the negative association of MV with life satisfaction, which should provide practical implications for future intervention and prevention efforts of mitigating the adverse effects of materialism on young individuals.

**Table 1:** Testing hypotheses of direct and indirect effects of the pathways.

Effects	Path coefficient (SE)	95% Confidence interval	
		LLCI	ULCI
Direct effects			
H1: MV → SE	−0.074^*^ (0.03)	−0.135	−0.013
H3: MV → SSR	−0.093^*^ (0.03)	−0.157	−0.029
H4: MV → OSR	−0.031 (0.03)	−0.091	0.029
Indirect effects			
H2: MV → SE → LS	ab = −0.016^*^	−0.029	−0.003
H5: MV → SSR → LS	ab = −0.011^*^	−0.021	−0.003
H6: MV → OSR → LS	ab = −0.002	−0.007	0.002
H7: MV → SE → LS _girls_ > MV → SE → LS _boys_	Δ*ab* = 0.007	−0.020	0.034

MV, materialistic values; SE, self-esteem; SSR, same-sex peer relationship; OSR, opposite-sex peer relationship; LS, life satisfaction. Gender differences in H5&H6 were tested without directional hypotheses considering the mixed findings in the extant literature. ^*^p < 0.05.

## Method

### Participants and procedure

The present study used data from the California Families Project, an ongoing longitudinal study of Mexican-origin youth (*N* = 674) and their parents. It involves secondary data analysis of the California Families Project (e.g., Cheng and Robins, 2026; Shi et al., 2024). No previous published work has used the project data to examine the underlying mechanisms for the association of materialistic values with life satisfaction. Sample size was determined prior to the project data collection.

Children were drawn at random from rosters of students from the Sacramento and Woodland, CA school districts, in 2006-07. The focal child had to be in the 5^th^ grade, of Mexican origin, and living with their biological mother in order to participate in the study. Approximately 72.6% of the eligible families agreed to participate in the study. The children (50% female) were assessed annually from age 10 to 19, and then at ages 21 and 23.

The present study was a longitudinal study, using data when the children were ages 12 (*M*_*age*_ = 12.81, *SD* = 0.49), 14 (*M*_*age*_ = 14.75, *SD* = 0.49), 16 (*M*_*age*_ = 16.80, *SD* = 0.51). Retention rates compared to the original sample are as follows: 86% (age 12), 90% (age 14), 89% (age 16). The data of children at Age 12 were included as the control variable for testing the mechanisms underlying the negative association of MV with life satisfaction (ages 14&16).

Participants were interviewed in their homes in Spanish or English, depending on their preference. Interviewers were all bilingual and most were of Mexican heritage. Median total household income was between $30,000 and $35,000 (overall range of income = < $5,000 to > $95,000). Regarding nativity status, 83.6% of mothers and 89.4% of fathers were 1^st^ generation, and 16.4% of mothers and 10.6% of fathers were either 2^nd^ or 3^rd^ generation.

### Ethics statement

The California Families project was granted approval by the University of California, Davis Institutional Review Board (Protocol Number 217484-21).

### Measures

#### Materialistic values

The Mexican American Cultural Values Scale (MACVS; Hernandez et al., 2026; Knight et al., 2011) was developed through focus groups of immigrant and U.S. born Mexican-origin adolescents and adults who identified values that they ascribed to Mexican and American culture. Children completed the MACVS at ages 12, 14, and 16. The Materialistic Values scale includes the following five items: “Money is the key to happiness”; “Owning a lot of nice things makes one very happy”; “The more money one has, the more respect they should get from others”; “The best way for a person to feel good about himself/herself is to have a lot of money”; and “Children should be taught that it is important to have a lot of money”. Ratings were made on a 4-point scale from 1 (*not at all*) to 4 (*very much*). We computed a latent variable of materialistic values at each wave with the five items as indicators. Reliabilities and descriptive statistics for MV scale are shown in Table 2.

**Table 2:** Descriptive statistics and reliability across measurement items for study variables.

Variables	Age 12		Age 14		Age 16	
	*M (SD)*	*α/ω*	*M (SD)*	*α/ω*	*M (SD)*	*α/ω*
Materialistic values	1.84 (0.63)	0.79/0.82	1.76 (0.60)	0.79/0.81	1.80 (0.59)	0.79/0.81
Self-esteem	-	-	3.04 (0.55)	0.83/0.86	2.98 (0.53)	0.80/0.83
Same-sex peer relationship	-	-	3.30 (0.50)	0.69/0.81	3.27 (0.47)	0.67/0.80
Opposite-sex peer relationship			3.34 (0.51)	0.62/0.72	3.30 (0.47)	0.62/0.77
Life satisfaction	*-*		4.18 (0.71)	-	4.16 (0.71)	-

M, mean; SD, standard deviation; α, alpha reliability; ω, omega total reliability.

#### Life satisfaction

To assess life satisfaction, we used a single-item measure of global life satisfaction (i.e., “How satisfied or dissatisfied are you with your life as a whole?”; Campbell et al., 1976; Geda et al., 2025). Children rated this item at ages 14, 16 using a 5-point Likert scale ranging from 1 (*completely dissatisfied*) to 5 (*completely satisfied*). Means and standard deviations are *M* =4.18, *SD* = 0.71 (age 14); *M* = 4.16, *SD* = 0.71 (age 16).

#### Self-esteem

To assess self-esteem at ages 14 and 16, we used a five-item scale (Marsh et al., 2005, 2022) as follows: “Overall, you have a lot to be proud of”; “Most things you do, you do well”; “Overall, most things you do turn out well”; “You can do things as well as most people”; “If you really try you can do almost anything you want to do”. Ratings were made on a 4-point scale from 1 (*not at all*) to 4 (*very much*). We computed a latent variable of self-esteem at each wave with the five items as indicators. Reliabilities and descriptive statistics for self-esteem scale are shown in Table 2.

#### Peer relationship

##### Same-sex peer relationship

To assess same-sex peer relationship at ages 14 and 16, we used a five-item scale (Marsh et al., 2005, 2022) as follows: “It is difficult to make friends with [same sex: girls/boys]”; “You make friends easily with [same sex: girls/boys]”; “Not many [same sex: girls/boys] like you”; “You do not get along very well with [same sex: girls/boys]”; “You make friends easily with members of your own sex”. Ratings were made on a 4-point scale from 1 (*not at all*) to 4 (*very much*). We computed a latent variable of same-sex peer relationship at each wave with the five items as indicators. Reliabilities and descriptive statistics for self-esteem scale are shown in Table 2.

##### Opposite-sex peer relationship

To assess opposite-sex peer relationship at ages 14 and 16, we used a four-item scale (Marsh et al., 2005, 2022) as follows: “You are not very popular with members of the opposite sex”; “You make friends easily with members of the opposite sex”; “You have lots of friends of the opposite sex”; “You do not get along very well with members of the opposite sex”. Ratings were made on a 4-point scale from 1 (*not at all*) to 4 (*very much*). We computed a latent variable of opposite-sex peer relationship at each wave with the four items as indicators. Reliabilities and descriptive statistics for self-esteem scale are shown in Table 2.

#### Household income

Household income was included as a control variable, considering that children from poor families were often more materialistic and placed more importance on financial success and wealth (e.g., Cohen and Cohen, 1996; Ku, 2015; Li et al., 2025) than their affluent counterparts. At the start of data collection, total annual household income was reported by the mothers using a 20-point ordinal response scale, with response options increasing in $5,000 increments (1 = “Less than $5,000”, 2 = “$5,000-$10,000, …, up to 20 = “95,000 or more”). Median total household income was $32,500.

In Table 3, means and correlations with confidence intervals for all variables based on the average scores are presented.

**Table 3:** Means, standard deviations, and correlations with confidence intervals.

Variable	*M*	*SD*	1	2	3	4	5	6	7	8	9	10	11	12
1. c03MV	1.84	0.63												
2. c05MVt	1.76	0.60	0.50^**^											
			[0.44, 0.56]											
3. c07MV	1.80	0.59	0.39^**^	0.47^**^										
			[0.31, 0.46]	[0.40, 0.53]										
4. c05SSR	3.30	0.50	−0.09^*^	−0.09^*^	−0.07									
			[−0.17, −0.01]	[−0.17, −0.01]	[−0.15, 0.01]									
5. c07SSR	3.27	0.47	−0.06	−0.06	−0.12^**^	0.51^**^								
			[−0.15, 0.02]	[−0.14, 0.02]	[−0.20, −0.04]	[0.45, 0.57]								
6. c05SE	3.04	0.55	−0.06	−0.06	−0.04	0.38^**^	0.29^**^							
			[−0.14, 0.03]	[−0.13, 0.02]	[−0.12, 0.04]	[0.31, 0.44]	[0.21, 0.36]							
7. c07SE	2.98	0.53	−0.08	−0.08	−0.08	0.18^**^	0.36^**^	0.52^**^						
			[−0.16, 0.01]	[−0.16, 0.00]	[−0.16, 0.00]	[0.10, 0.26]	[0.29, 0.43]	[0.45, 0.57]						
8. c05OSR	3.34	0.51	−0.06	−0.01	−0.04	0.41^**^	0.24^**^	0.33^**^	0.24^**^					
			[−0.14, 0.03]	[−0.09, 0.07]	[−0.12, 0.04]	[0.34, 0.48]	[0.17, 0.32]	[0.25, 0.40]	[0.16, 0.32]					
9. c07OSR	3.30	0.47	−0.04	−0.05	−0.09^*^	0.34^**^	0.39^**^	0.30^**^	0.39^**^	0.54^**^				
			[−0.13, 0.04]	[−0.13, 0.03]	[−0.17, −0.01]	[0.26, 0.41]	[0.32, 0.45]	[0.22, 0.37]	[0.31, 0.45]	[0.48, 0.60]				
10. c05LS	4.18	0.71	0.00	−0.06^†^	−0.03	0.27^**^	0.18^**^	0.35^**^	0.24^**^	0.17^**^	0.15^**^			
			[−0.08, 0.09]	[−0.14, 0.02]	[−0.11, 0.05]	[0.19, 0.34]	[0.10, 0.26]	[0.28, 0.42]	[0.16, 0.32]	[0.09, 0.24]	[0.07, 0.23]			
11. c07LS	4.16	0.71	0.03	0.00	−0.04	0.13^**^	0.18^**^	0.19^**^	0.34^**^	0.04	0.12^**^	0.33^**^		
			[−0.06, 0.11]	[−0.08, 0.09]	[−0.12, 0.04]	[0.05, 0.21]	[0.11, 0.26]	[0.12, 0.27]	[0.26, 0.41]	[−0.04, 0.12]	[0.04, 0.20]	[0.25, 0.40]		
12. Income	7.29	4.26	−0.11^*^	−0.08	0.01	0.12^**^	0.06	0.13^**^	0.07	0.03	−0.05	0.11^*^	0.07	
			[−0.20, −0.03]	[−0.16, 0.01]	[−0.07, 0.10]	[0.04, 0.20]	[−0.02, 0.14]	[0.05, 0.22]	[−0.02, 0.15]	[−0.06, 0.11]	[−0.13, 0.04]	[0.03, 0.19]	[−0.02, 0.15]	
13. Gender	0.50	0.50	−0.15^**^	−0.18^**^	−0.18^**^	−0.04	−0.00	−0.06	−0.04	−0.07	−0.10^*^	−0.09^*^	−0.05	−0.03
			[−0.23, −0.07]	[−0.25, −0.10]	[−0.26, −0.10]	[−0.12, 0.04]	[−0.08, 0.08]	[−0.14, 0.02]	[−0.12, 0.04]	[−0.14, 0.01]	[−0.18, −0.02]	[−0.17, −0.02]	[−0.13, 0.03]	[−0.11, 0.05]

MV, materialistic values; SE, self-esteem; SSR, same-sex peer relationship; OSR, opposite-sex peer relationship; LS, life satisfaction. ^*^p < 0.05, ^**^p < 0.01,^†^*p* < 0.10.

### Data analysis plan

All data cleaning and analyses were conducted in R (Posit team, 2023; RStudio Team, 2021) using full information maximum likelihood estimation (FIML) to account for missing data in endogenous variables (Enders and Bandalos, 2001; Tang and Tong, 2025; Zhang and Savalei, 2023). We used the following R packages (Wickham and Bryan, 2023) to analyze the data and visualize the results: *psych* (Revelle, 2020; Waller and Revelle, 2025), and *lavaan* (Rosseel, 2012; Tang et al., 2024). Model fit was assessed via comparative fit index (CFI) and root-mean-square error of approximation (RMSEA). We interpreted good fit as values greater than or equal to 0.90 for CFI (Bentler, 1990; Gomez et al., 2024; Goretzko et al., 2024; Tang et al., 2024; Zhang and Savalei, 2023) and less than or equal to 0.08 for RMSEA (Browne and Cudeck, 1993; Gomez et al., 2024; Tang et al., 2024; Zhang and Savalei, 2023). We compared models based on a holistic evaluation of multiple criteria: difference in CFI above the threshold of 0.01, difference in RMSEA above the threshold of 0.015, and chi-square difference test (Chen, 2007; Cheung and Rensvold, 2002; Gomez et al., 2024; Tang et al., 2024).

#### Longitudinal measurement invariance analyses

We tested longitudinal measurement invariance for all latent variables across waves by comparing four nested models: (1) a *configural* model, which restricted equal factor structures (1-factor model) across waves; (2) a *weak invariance* model, which constrained equal factor loadings; (3) a *strong invariance* model, which constrained equal factor loadings and intercepts; and (4) a *strict invariance* model, which restricted equal residual variances on the basis of the lesser constrained model.

Each item was specified to correlate to itself across waves. Residual correlations were permitted between select item pairs based on modification indices and theoretical rationale: the second (“Most things you do, you do well.”) and third items (“Overall, most things you do turn out well.”) of the self-esteem scale, the first (“It is difficult to make friends with [SAME SEX: girls/boys]”) and the second items (“You make friends easily with [SAME SEX: girls/boys]”), and the third (“Not many [SAME SEX: girls/boys] like you.”) and fourth items (“You do not get along very well with [SAME SEX: girls/boys].”) of the same-sex peer relationship scale.

If more constrained models showed comparable or better fit than less constrained ones, we concluded that the level of measurement invariance corresponding to the more constrained model was established. When the strong invariance model fit was significantly worse than the weak model, we tested a partial strong invariance model (freeing selected intercepts) to retain as much invariance as possible (Protzko, 2025; Widaman et al., 2010). If partial strong invariance held, we proceeded to test strict invariance.

The following item intercepts were allowed to be time-varying based on modification indices and theoretical rationale: the first item (“Money is the key to happiness”) of the materialistic value scale, the second item (“You make friends easily with [SAME SEX: girls/boys]”) of the same-sex peer relationship scale, and the first (“You are not very popular with members of the opposite sex”) and third items (“You have lots of friends of the opposite sex”) of the opposite-sex peer relationship. For model identification, all factor variances were fixed to 1 across waves.

#### Hypotheses test for mediation of SE, SSR, and OSR in the MV-life satisfaction relation

To test the total effect of materialistic values (MV) on life satisfaction, we fitted a model with autoregressive paths and concurrent paths from MV to life satisfaction. To evaluate potential mediating mechanisms, we estimated three separate models, each examining self-esteem (SE), same-sex peer relationship (SSR), or opposite-sex peer relationship (OSR) as mediators. Measurement models were unchanged from their respective optimal longitudinal invariance models. In all models, equality constraints were imposed on autoregressive and concurrent mediation paths across waves. Mediation was tested by evaluating (1) the *a* path (MV → mediator) and *b* path (mediator → life satisfaction) individually, and (2) their joint indirect effect using Monte Carlo confidence intervals for the product term (*a*×*b*). If multiple mediators showed significant effects, we constructed a comprehensive model including all significant mediators simultaneously to examine unique effects while controlling for alternative pathways. Additionally, we replicated mediation tests by including household income as a covariate for MV, mediators, and life satisfaction in order to control for the potential confounding effect of this variable.

#### Hypotheses test for moderation of gender

We tested the moderating effects of gender on the mediation paths via multi-group model analyses. First, we tested measurement invariance across gender groups using procedures similar to those described in the *Longitudinal Measurement Invariance Analyses* section, but with equity constraints applied across both measurement waves and gender groups. Second, based on the optimal measurement invariance model, we fitted longitudinal mediation models with either self-esteem or peer relationship as the mediator via multi-group model, constraining the *a* (MV → mediator) and *b* (mediator → life satisfaction) paths to be equal across gender groups. Third, we tested for gender moderation by relaxing the equality constraints on the mediation paths and evaluating group differences in indirect effects using the Delta method and Monte Carlo method (MacKinnon et al., 2004; Pigart et al., 2024), followed by model comparisons between the constrained and unconstrained mediation models.

## Results

Tables 2, 3 present the Cronbach's alpha (Castro-Alvarez et al., 2026; Cronbach, 1951; Gomez et al., 2024), McDonald's Omega (Dunn et al., 2024; Gomez et al., 2024; McDonald, 1999), means (Cohen et al., 2003; Gomez et al., 2024), standard deviations (SD; Cohen et al., 2003; Gomez et al., 2024), and intercorrelations for all variables based on the average scores for the corresponding scales. The 2-year and 4-year lagged autocorrelations for materialistic values (MV) ranged from 0.39 to 0.50, indicating moderate rank-order stability. Self-esteem (SE) showed a high rank-order stability of 0.51 over the 2-year period.

Missing data rates across all indicators and measurement time points ranged from 0% to 12%, which is well within the robust performance missing rates for FIML estimation (Enders and Bandalos, 2001; Tang and Tong, 2025). The complete item-level missing-ness report and the corresponding R Markdown script are available at https://osf.io/tbc27?view_only=88e8e428fd6d4e2c914b1467fc37247e.

### Longitudinal measurement invariance for MV, SE, SSR, and OSR

We tested longitudinal measurement invariance for materialistic values (MV; Hernandez et al., 2026) in relation to self-esteem (SE), same-sex peer relationship (SSR), and opposite-sex peer relationship (OSR) using a series of nested models. As described earlier, for each construct, we evaluated configural (equal forms), weak (equal loadings), strong (partial equal intercepts), and strict (equal error variances) invariance.

MV and SE demonstrated strict invariance (Table 4), supported by model fit comparisons (χ^2^/df < 2, ΔCFI < 0.010, ΔRMSEA < 0.010). The configural model (Figure 1) provided a good baseline fit (CFI = 0.974, RMSEA = 0.029), and subsequent invariance constraints did not significantly degrade model fit (strict invariance: CFI = 0.963, RMSEA = 0.032). We retained the strict invariance model for parsimony.

**Table 4:** Model comparisons for longitudinal measurement invariance.

Model	χ^2^	*df*	*p*	CFI	|ΔCFI|	RMSEA [90%CI]	|ΔRMSEA|
Child self-esteem and materialistic values							
Configural	383.13	248		0.974		0.029 [.023, 0.035]	
Weak	395.74	263	0.632	0.974	0.000	0.028 [.022, 0.034]	0.001
Partial strong	449.91	276	< 0.001	0.966	0.008	0.031 [.026, 0.037]	0.003
Strong	474.17	278	< 0.001	0.962	0.004	0.033 [.028, 0.038]	0.002
**Strict**	**480.71**	**291**	**0.001**	**0.963**	**0.003**	**032 [.027, 0.037]**	**0.001**
Child same-sex peer relationship and materialistic values							
Configural	413.49	244		0.963		0.033 [.027, 0.038]	
Weak	428.00	259	1.000	0.963	0.000	0.032 [.027, 0.037]	0.001
Partial strong	477.39	271	< 0.001	0.955	0.008	0.035 [.029, 0.040]	0.003
Strong	516.76	274	< 0.001	0.947	0.008	0.037 [.032, 0.042]	0.002
**Strict**	**514.03**	**286**	**0.001**	**0.950**	**0.003**	**035 [.031, 0.040]**	**0.000**
Child opposite-sex peer relationship and materialistic values							
Configural	383.21	206		0.959		0.037 [.031, 0.042]	
Weak	393.38	220	0.734	0.959	0.000	0.035 [.030, 0.041]	0.002
Partial strong	441.56	230	< 0.001	0.951	0.008	0.038 [.033, 0.043]	0.003
Strong	492.15	234	< 0.001	0.940	0.011	0.042 [.037, 0.047]	0.004
**Strict**	**482.18**	244	**< 0.001**	**0.944**	**0.006**	**039 [.034, 0.044]**	**0.001**

χ^2^, Chi-square test statistics; *df*, degrees of freedom; *p, p*-value for Chi-square difference test; CFI, comparative fit index; |ΔCFI|, difference in CFI of the adjacent models; RMSEA, root-mean-square error of approximation; 90%CI, 90% confidence interval; |ΔRMSEA|, difference in RMSEA of adjacent models. Models in bolded text are retained. Strict invariance model was compared with partial strong invariance model.

**Figure 1:**
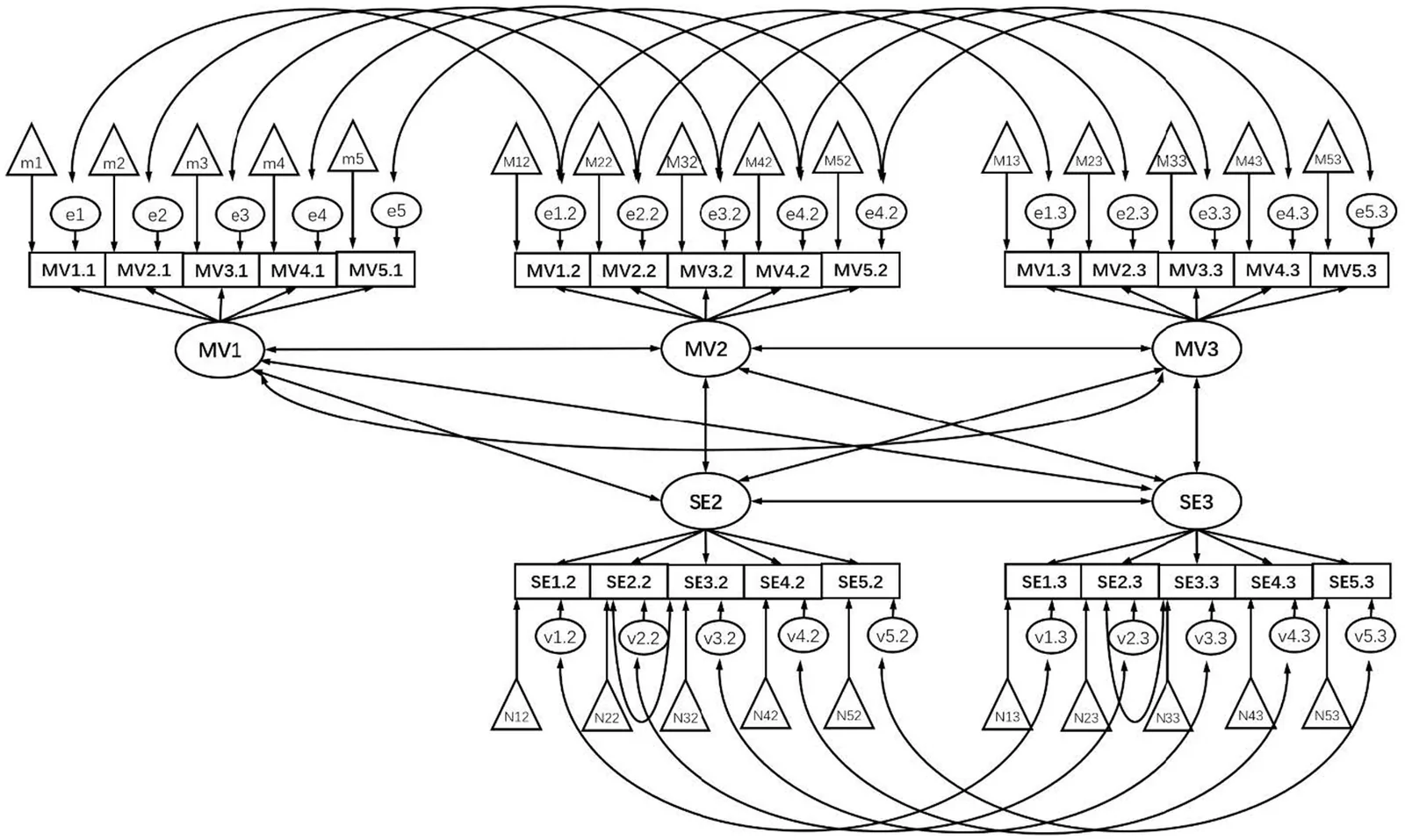
Model for longitudinal measurement invariance analysis.

MV and SSR also met strict invariance criteria (Table 4). The configural model showed acceptable fit (CFI = 0.963, RMSEA = 0.033), and invariance constraints yielded minimal changes in fit indices (strict invariance: CFI = 0.950, RMSEA = 0.035). The strict model was retained.

Similarly, MV and OSR exhibited strict invariance (Table 4). The configural model had adequate fit (CFI = 0.959, RMSEA = 0.037), and imposing invariance constraints did not substantially worsen fit (strict invariance: CFI = 0.944, RMSEA = 0.039). The strict model was again preferred for parsimony.

### Longitudinal mediating effects of SE, SSR, and OSR on the relation between MV and LS

#### Total effect of materialistic values

The model examining the total effect of materialistic values (MV) on life satisfaction (LS) demonstrated acceptable fit (χ^2^ = 289.23, *df* = 136, CFI = 0.947, RMSEA = 0.042, 95%CI = [0.035, 0.049]). After controlling for lag-1 autoregressive effects, higher MV at one wave marginally predicted lower concurrent life satisfaction (*c* = −0.034, 95%CI = [−0.072, 0.005], *z* = −1.724, *p* = 0.085).

#### Self-esteem as mediator

The model testing self-esteem (SE) as a mediator (Figure 2A) demonstrated acceptable fit (χ^2^ = 755.68, *df* = 373, CFI = 0.931, RMSEA = 0.040, 95%CI = [0.036, 0.044]). After controlling for lag-1 autoregressive effects, higher MV at one wave significantly predicted lower self-esteem (SE) at the same wave (*a* = −0.074, *p* = 0.017, 95%CI = [−0.135, −0.013], Table 5), which was consistent with H1, and in turn, predicted lower life satisfaction (LS) at the same wave (*b* = 0.211, *p* < 0.001, 95%CI = [0.171, 0.250]). Consistent with H2, the indirect effect, tested using the Monte Carlo method, was significant, *ab* = −0.016, 95% CI = [−0.029, −0.003], with an effect size of $abc^{'}=\frac{-0.074\times 0.211}{-0.014}\approx 1.11$. This indirect effect remained significant after controlling for household income, *ab* = −0.015, 95%CI = [−0.028, −0.002].

**Figure 2:**
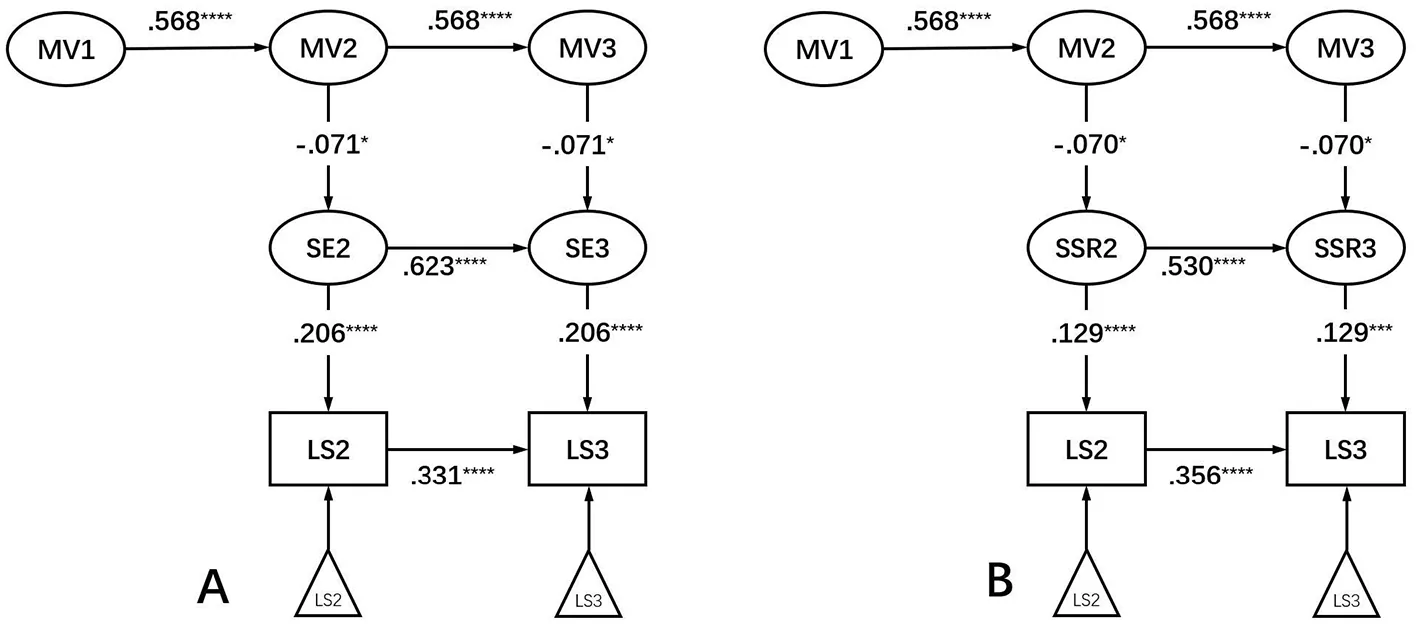
Structural model for the model to test the mediating effects of self-esteem and same-sex peer relationship on materialistic values and life satisfaction. Nonsignificant paths and covariate income were not displayed. Figure **(A)** is the left panel, and figure **(B)** is the right panel. MV, materialistic values; SE, self-esteem; SSR, same-sex peer relationship; LS, life satisfaction. ^*^p < 0.05, ^**^p < 0.01, ^***^p < 0.001.

**Table 5:** Model statistics from models testing self-esteem and peer relationship as mediators of materialistic values and life satisfaction.

Model	Without the covariate			With the covariate		
	*b* (SE)	z	*p*	*b* (SE)	z	*p*
Self-esteem as mediator						
**Autoregression**						
MV t-1 → MV t	**0.55** (0.03)	16.87	< 0.001	**0.56** (0.03)	16.56	< 0.001
Self-Esteem t-1 → Self-Esteem t	**0.56** (0.05)	11.69	< 0.001	**0.62** (0.05)	12.30	< 0.001
Life Satisfaction t-1 → Life Satisfaction t	**0.33** (0.03)	12.04	< 0.001	**0.33** (0.03)	11.65	< 0.001
**Regression**						
MV t → Self-Esteem t (*a* path)	**−0.07** (0.03)	−2.38	0.017	**−0.07** (0.03)	−2.20	0.028
Self-Esteem t → Life Satisfaction t (*b* path)	**0.21** (0.02)	10.53	< 0.001	**0.21** (0.02)	10.04	< 0.001
MV t → Life Satisfaction t (*c*′ path)	−0.01 (0.02)	−0.77	0.441	−0.01 (0.02)	−0.44	0.663
Same-sex peer relationship as mediator						
**Autoregression**						
MV t-1 → MV t	**0.55** (0.03)	16.86	< 0.001	**0.57** (0.03)	16.54	< 0.001
Same-Sex Peer t-1 → Same-Sex Peer t	**0.53** (0.06)	8.90	< 0.001	**0.53** (0.06)	8.41	< 0.001
Life Satisfaction t-1 → Life Satisfaction t	**0.36** (0.03)	12.81	< 0.001	**0.36** (0.03)	12.41	< 0.001
**Regression**						
MV t → Same-Sex Peer t (*a* path)	**−0.09** (0.03)	−2.86	0.004	**−0.07** (0.03)	−2.11	0.035
Same-Sex Peer t → Life Satisfaction t (*b* path)	**0.12** (0.02)	5.46	< 0.001	**0.13** (0.02)	5.67	< 0.001
MV t → Life Satisfaction t (*c*′ path)	−0.02 (0.02)	−1.05	0.661	−0.02 (0.02)	−0.81	0.416
Opposite-sex peer relationship as mediator						
**Autoregression**						
MV t-1 → MV t	**0.55** (0.03)	16.89	< 0.001	**0.57** (0.03)	16.58	< 0.001
Opposite-Sex Peer t-1 → Opposite-Sex Peer t	**0.57** (0.05)	12.40	< 0.001	**0.56** (0.05)	11.59	< 0.001
Life Satisfaction t-1 → Life Satisfaction t	**0.37** (0.03)	13.33	< 0.001	**0.37** (0.03)	12.77	< 0.001
**Regression**						
MV t → Opposite-Sex Peer t (*a* path)	−0.03 (0.03)	−1.00	0.317	−0.02 (0.03)	−0.51	0.607
Opposite-Sex Peer t → Life Satisfaction t (*b* path)	0.02 (0.02)	0.80	0.425	0.02 (0.02)	0.92	0.356
MV t → Life Satisfaction t (*c*′ path)	0.00 (0.02)	0.17	0.869	−0.00 (0.02)	−0.23	0.817

*P* value ≤ 0.05 was deemed as significant. The bold values represent significant findings.

#### Same-sex peer relationship as mediator

The model for same-sex peer relationship (SSR; Figure 2B) also showed acceptable fit (χ^2^ = 732.15, *df* = 368, CFI = 0.925, RMSEA = 0.039, 95%CI = [0.035, 0.043]). Consistent with H3, higher MV predicted lower SSR concurrently (*a* = −0.093, *p* = 0.004, 95%CI = [−0.157, −0.029], Table 5). Moreover, lower SSR predicted reduced LS at the same wave (*b* = 0.120, *p* = < 0.001, 95%CI = [0.077, 0.163]). Consistent with H5, The indirect effect was significant (*ab* = −0.011, 95% Monte Carlo CI = [−0.021, −0.003], effect size $abc^{'}=\frac{-0.093\times 0.120}{-0.020}\approx 0.55$), and remained significant after income adjustment, *ab* = −0.009, 95% Monte Carlo CI = [−0.019, −0.001].

#### Opposite-sex peer relationship as mediator

For opposite-sex peer relationship (OSR), model fit was acceptable (χ^2^ = 667.351, *df* = 320, CFI = 0.921, RMSEA = 0.041, 95%CI = [0.037, 0.04]). MV was not associated with OSR (*a* = −0.031, *p* = 0.316, 95%CI = [−0.091, 0.029], Table 5), which was inconsistent with H4. Moreover, inconsistent with H6, MV was not indirectly associated with LS via OSR (*b* = 0.073, *p* = < 0.001, *ab* = −0.002, 95% CI = [−0.007, 0.002], effect size $abc^{'}=\frac{-0.030\times 0.073}{-0.030}\approx 0.07$). Income adjustment did not alter these null findings (*ab* = −0.001, 95%CI = [−0.007, 0.003]).

#### Self-esteem and same-sex peer relationship as mediator

To rule out the influences of correlated mediators, we fitted a combined model including both SE and SSR as mediators (Figure 3). The model fit was acceptable, χ2 = 1311.96, df = 685, CFI = 0.918, RMSEA = 0.038, 95%CI = [0.035, 0.041]. When both mediators were included, only SE showed a significant indirect effect (*ab* = −0.017, 95% Monte Carlo CI = [−0.031, −0.003]), whereas the mediating effect of SSR reduced to be non-significant (*ab* = 0.001, 95% Monte Carlo CI = [−0.006, 0.007]). A direct comparison confirmed SE's stronger mediation (Δ*ab* = −0.017, 95% Monte Carlo CI = [−0.033, −0.024],*p* = 0.039). Controlling for household income did not alter these patterns: SE remained significant (*ab* = −0.015, 95% Monte Carlo CI = [−0.029, −0.002]) SSR remained non-significant (*ab* = −0.001, 95% Monte Carlo CI = [−0.006, 0.004]), and the mediation difference, though slightly attenuated, persisted (Δ*ab* = −0.014, 95% Monte Carlo CI = [−0.029, −0.014], *p* = 0.067).

**Figure 3:**
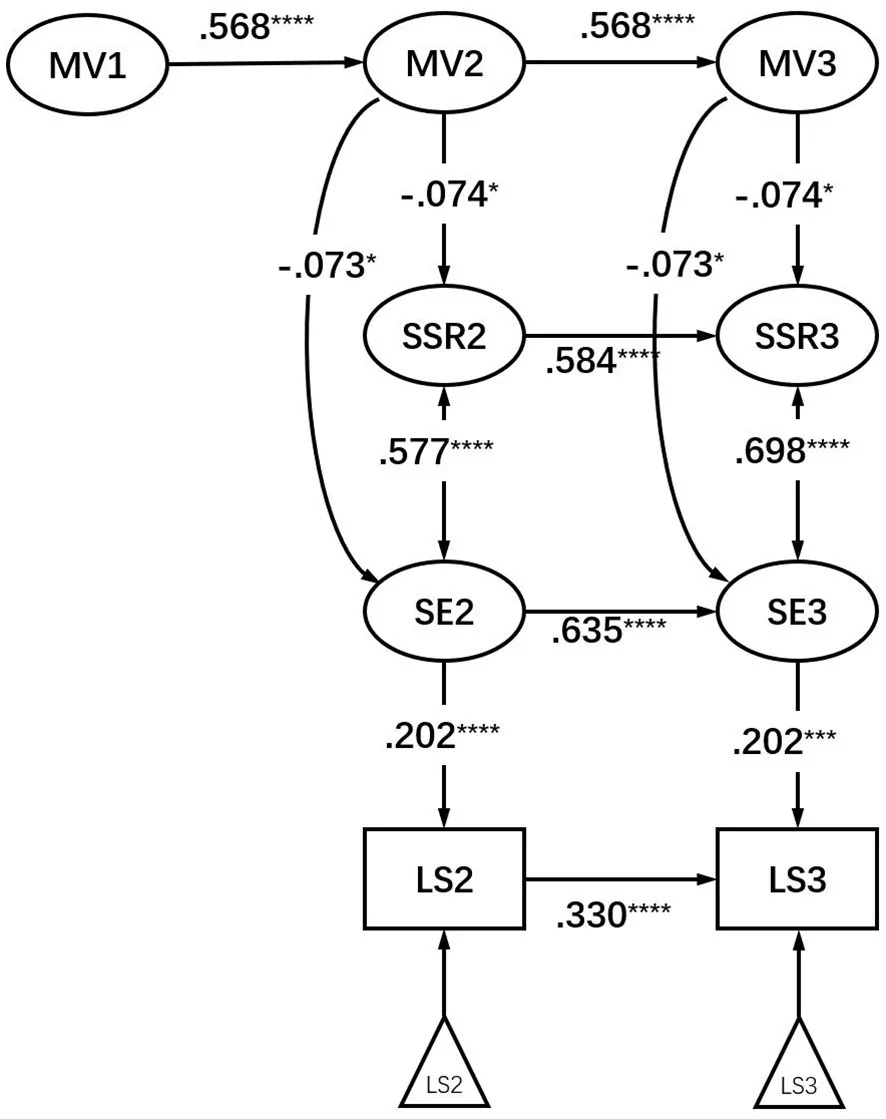
Structural Model for the Cross-lagged Panel Model to test Simultaneously the Mediating Effects of Self-Esteem and Same-Sex Peer relationship on Materialistic Values and Life Satisfaction. Non-significant paths and covariate income were not displayed. MV, materialistic values; SE, self-esteem; SSR, same-sex peer relationship; LS, life satisfaction. ^*^p < 0.05, ^**^p < 0.01, ^***^p < 0.001.

#### Moderating effect of gender

Before evaluating gender differences in latent variables, we first tested measurement invariance of self-esteem (SE), same-sex peer relationship (SSR), and materialistic values (MV) across gender groups using multi-group model analyses. MV and SE demonstrated weak invariance (Table 6; χ^2^/df < 2, ΔCFI < 0.010, ΔRMSEA < 0.010). We did not establish a stronger invariance due to three considerations: (1) ΔCFI's relative robustness to sample size and model size (Cheung and Rensvold, 2002; Kalkbrenner and Lenz, 2026; Meade et al., 2008); (2) minimal improvement potential from releasing intercept equality constraints based on score tests; (3) similar moderation results across weak and partial strong invariance models. The configural model (Figure 1) provided a good baseline fit (CFI = 0.962, RMSEA = 0.035), and metric invariance constraints did not significantly degrade model fit (CFI = 0.958, RMSEA = 0.035). We retained the weak invariance model for parsimony. Similarly, MV and SSR also showed weak invariance (Table 6). The configural model showed acceptable fit (CFI = 0.953, RMSEA = 0.038), and factor loading constraints yielded minimal changes in fit indices (CFI = 0.940, RMSEA = 0.040). The weak invariance model was retained.

**Table 6:** Model comparisons for longitudinal measurement invariance across gender groups.

Model	χ^2^	*df*	*p*	CFI	|ΔCFI|	RMSEA [90%CI]	|ΔRMSEA|
Self-esteem and materialistic values							
Configural	689.80	496		0.962		0.035 [0.029, 0.041]	
**Weak**	**749.10**	**536**	**0.025**	**0.958**	**0.004**	**0.035 [0.029, 0.041]**	**0.000**
Partial Strong	846.56	565	< 0.001	0.944	0.014	0.040 [0.034, 0.045]	0.005
Strong	899.27	576	< 0.001	0.936	0.007	0.042 [0.037, 0.047]	0.002
Strict	949.45	605	< 0.001	0.932	0.012	0.042 [0.037, 0.047]	0.002
Same-sex relationship and materialistic values							
Configural	714.69	488		0.950		0.038 [0.032, 0.044]	
**Weak**	**801.76**	**528**	**< 0.001**	**0.940**	**0.010**	**0.040 [0.035, 0.046]**	**0.002**
Partial Strong	891.02	557	< 0.001	0.926	0.014	0.043 [0.038, 0.049]	0.003
Strong	956.37	568	< 0.001	0.914	0.012	0.046 [0.041, 0.051]	0.003
Strict	990.62	597	< 0.001	0.913	0.013	0.046 [0.040, 0.050]	0.003

χ^2^, Chi-square test statistics; *df*, degrees of freedom; *p, p*-value for Chi-square difference test; CFI, comparative fit index; |ΔCFI, difference in CFI of the adjacent models; RMSEA, root-mean-square error of approximation; 90%CI, 90% confidence interval; |ΔRMSEA|, difference in RMSEA of adjacent models. Models in bolded text are retained. Strict invariance model was compared with partial strong invariance model.

The multi-group model with self-esteem (SE) mediation showed acceptable fit (χ^2^ =1128.71, *df* = 706, CFI = 0.923, RMSEA = 0.043, 95%CI = [0.038, 0.048]). For girls, SE significantly mediated the relationship between MV and life satisfaction (*ab* = −0.023, 95% Monte Carlo CI = [−0.045, −0.002], effect size $abc^{'}$ 1.42). Specifically, higher MV predicted lower SE at the same wave (*a* = −0.105, *p* = 0.034, 95%CI = [−0.202, −0.008], Table 6), which then predicted lower life satisfaction at the same wave (*b* = 0.216, *p* < 0.001, 95%CI = [0.162, 0.270], Table 6). This remained significant after controlling for household income, *ab* = −0.025, 95%CI = [−0.048, −0.005]. For boys, the mediation was also significant (*ab* = −0.016, 95% Monte Carlo CI = [−0.033, −0.001], effect size $abc^{'}=$ 1.01); higher MV predicted lower SE (*a* = −0.085, *p* = 0.038, 95%CI = [−0.160, −0.005], Table 7), which then predicted lower life satisfaction at the same wave (*b* = 0.197, *p* < 0.001, 95%CI = [0.142, 0.250]). This mediation became non-significant after income adjustment, *ab* = −0.013, 95%CI = [−0.030, 0.003], suggesting the less robustness of SE mediation among boys. Inconsistent with H7, group differences in indirect effects were non-significant (income unadjusted: Δ*ab* = 0.007, SE = 0.014, *p* = 0.631, 95% Monte Carlo CI = [−0.020, 0.034], Δχ^2^ = 0.388, *df* = 2, *p* = 0.823; income adjusted: Δ*ab* = 0.013, SE = 0.014, *p* = 0.366, 95% Monte Carlo CI = [−0.015, 0.040], Δχ^2^ = 0.871, *df* = 2, *p* = 0.647), suggesting that the moderating effect of gender group was not statistically significant.

**Table 7:** Model statistics from multi-group models testing gender as a moderator on the mediation of self-esteem and same-sex peer relationship.

Model	Without the covariate			With the covariate		
	*b* (SE)	z	*p*	*b* (SE)	z	*p*
Self-esteem as mediator						
**Boys**						
MV t → Self-Esteem t (*a* path)	–**0.08** (0.04)	−2.08	0.038	−0.06 (0.04)	−1.52	0.128
Self-Esteem t → Life Satisfaction t (*b* path)	**0.20** (0.04)	7.01	< 0.001	**0.21** (0.03)	7.30	< 0.001
MV t → Life Satisfaction t (*c*′ path)	−0.02 (0.02)	−0.84	0.399	−0.01 (0.02)	−0.30	0.767
Indirect Effect (*ab*)	**−0.02** (0.01)	−2.01	0.045	−0.01 (0.01)	−1.49	0.135
**Girls**						
MV t → Self-Esteem t (*a* path)	**−0.11** (0.05)	−2.12	0.034	**−0.12** (0.05)	−2.42	0.015
Self-Esteem t → Life Satisfaction t (*b* path)	**0.22** (0.03)	7.87	< 0.001	**0.21** (0.03)	7.30	< 0.001
MV t → Life Satisfaction t (*c*′ path)	−0.02 (0.02)	−0.84	0.399	−0.01 (0.02)	−0.30	0.767
Indirect Effect (*ab*)	**−0.02** (0.01)	−2.06	0.040	**−0.03** (0.01)	−2.31	0.021
Same-sex peer relationship as mediator						
**Boys**						
MV t → Same-Sex Peer t (*a* path)	**−0.09** (0.04)	−2.13	0.033	−0.06 (0.04)	−1.48	0.145
Same-Sex Peer t → Life Satisfaction t (*b* path)	**0.12** (0.03)	3.78	< 0.001	**0.12** (0.03)	3.48	< 0.001
MV t → Life Satisfaction t (*c*′ path)	−0.02 (0.02)	−1.26	0.208	−0.02 (0.02)	−0.85	0.396
Indirect Effect (*ab*)	−0.01 (0.01)	−1.87	0.061	−0.01 (0.01)	−1.35	0.176
**Girls**						
MV 1 → Same-Sex Peer t (*a* path)	**−0.12** (0.05)	−2.23	0.026	−0.10 (0.05)	−1.87	0.062
Same-Sex Peer t → Life Satisfaction t (*b* path)	**0.11** (0.03)	3.63	< 0.001	**0.12** (0.03)	4.04	< 0.001
MV t → Life Satisfaction t (*c*′ path)	−0.02 (0.02)	−1.26	0.208	−0.02 (0.02)	−0.85	0.396
Indirect Effect (*ab*)	−0.01 (0.01)	−1.87	0.062	−0.01 (0.01)	−1.66	0.097

*P* value ≤ 0.05 was deemed as significant. The bold values represent significant findings.

The same-sex peer relation (SSR) longitudinal mediation model also showed acceptable fit (χ^2^ = 1109.83, *df* = 696, CFI = 0.914, RMSEA = 0.045, 95%CI = [0.038, 0.048]). SSR mediation was significant for both genders (boys: *ab* = −0.010, SE = 0.006, *p* = 0.061, 95% Monte Carlo CI = [−0.023, −0.001], effect size $abc^{'}=$ 0.43; girls: *ab* = −0.012, SE = 0.007, *p* = 0.062, 95% Monte Carlo CI = [−0.028, −0.001], effect size $abc^{'}=$ 0.52) and became non-significant after income adjustment (boys: *ab* = −0.007, 95% Monte Carlo CI = [−0.019, 0.002], effect size $abc^{'}=$ 0.42; girls: *ab* = −0.012, 95% Monte Carlo CI = [−0.029, 0.001], effect size $abc^{'}=$ 0.72). Gender differences in indirect effects were non-significant (income unadjusted: Δ*ab* = 0.002, SSR = 0.008, *p* = 0.811, 95% Monte Carlo CI = [−0.015, 0.020], Δχ^2^ = 0.386, *df* = 2, *p* = 0.824; income adjusted: Δ*ab* = 0.005, SSR = 0.009, *p* = 0.578, 95% Monte Carlo CI = [−0.012, 0.024], Δχ^2^ = 0.380, *df* = 2, *p* = 0.827).

## Discussion

Based on the self-determination theory (Grassinger et al., 2024; Ryan and Deci, 2000), the present study is among the first few to examine the underlying processes that account for the negative linkages between materialistic values and life satisfaction. With recruitment of Mexican-origin adolescents, findings indicate that this association is mediated by self-esteem: Higher MV in adolescence act as a significant predictor of lower self-esteem, which in turn leads to lower life satisfaction. Same- but not opposite-sex peer relationship is an alternative mediator for exaplaining the association of MV with life satisfaciton. Compared to same-sex peer relationship, self-esteem is more substantial in explaining the association of MV with life satisfaction. Mediating effects of self-esteem and same-sex peer relationship are found similar across gender.

The negative linkage of MV with life satisfaction among Mexican-origin adolescents replicates the long-established pattern between MV and life satisfaction (Bradshaw et al., 2023; Moldes et al., 2025). Note that rather few studies have directly examined the underlying mechanisms for this negative linkage, except one study by Santos and Cassidy (2024), in which self-compassion, a variable describing how compassionate we are toward ourselves and how non-judgmental to our inadequacies and failures (Herrera and Turk, 2025; Neff, 2011), has been reported as a significant mediator. As compared to self-compassion which involves coping strategies and orients toward behavioral/cognitive styles (Herrera and Turk, 2025), self-esteem is a more fundamental characteristic about self (Muris and Otgaar, 2023). Findings in this study contribute to the literature by indicating the significant mediating role of self-esteem in explaining the negative association of MV with life satisfaction. Moreover, serial mediating effects of self-esteem and self-control are identified for the negative association of MV with a behavioral outcome—problematic social network sites use (Wang et al., 2024). These findings (Wang et al., 2024) validate the more fundamental role of self-esteem as compared to other self-related characteristics in explaining the adverse effects of materialistic values endorsement on adolescents.

In the present study, same-sex peer relationship is found to be an alternative, significant mediator. This finding confirms our hypothesis based on previous findings (Banerjee and Dittmar, 2008; Dunkeld et al., 2020; Moldes, 2025) indicating that higher MV are associated with poorer interpersonal relationships, which further detriment life satisfaction (Hall et al., 2023). Note that research (e.g., Harris and Orth, 2020; Pan et al., 2024) indicates that self-esteem and social relationship have a positive, reciprocal link, mutually contributing to each other across the lifespan. Accordingly, we examine the two mediators (i.e., self-esteem and same-sex peer relationship) in parallel in the same model, in order to disentangle the specific contribution of each mediator (Coutts and Hayes, 2023; Preacher and Hayes, 2008). Findings indicate that the mediating effect of self-esteem is significant, but not the effect of same-sex peer relationship, demonstrating that, as compared to same-sex peer relationship, self-esteem plays a more substantial role and more closely resonates with the adverse effect of materialistic values endorsement on adolescent life satisfaction.

## Theoretical implications

This research makes several theoretcial contributions. First, it demonstrates the crucial role of self-esteem in explaining how higher MV are related to lower life satisfaction. Findings validate that high materialistic values escalate low self-esteem. Altogether, an individual's pursuit of external material goods likely comes with a “price”, as he/she holds a lower level of self-esteem with the pursuit. Combined with research indicating that individuals with high materialistic values view possessions as a means of compensating for negative self-concepts (Chang et al., 2024; Chaplin and John, 2010) and of constructing a more positive self (Dittmar and Isham, 2022; Richins and Dawson, 1992), the present study suggests a vicious cycle of high materialistic values and low self-esteem in actuality.

This research furthermore provides evidence that low self-esteem accounts for the negative association of high MV with low life satisfaction. In particular, with the models disentangling the impact of self-esteem from inter-personal relationships (i.e., same-sex peer relationship), the estimated effect of self-esteem still remains strong whereas the mediating effect of same-sex peer relationship reduces to be non-significant, highlighting self-esteem as the primary mechanism accounting for the association of endorsement of materialistic values with low life satisfaction. Overall, by understanding the cognitive mechanisms that link materialism, self-esteem, and life satisfaction, this research contributes to deepening our knowledge of how materialistic values, which are increasingly being promoted at a societal level, shape young individuals' self-esteem, and thereby, lays the ground for interventions aimed at counteracting the adverse effects of materialism on life satisfaction.

From an inter-personal perspective, this research contributes to the literature by identifying same-sex peer relationship as an alternative pathway through which materialistic values influence life satisfaction. Materialism undermines same-sex peer relationship during adolescence, thereby reducing adolescent life satisfaction. These findings highlight the ineffectiveness of constructing one's identity among peers through material acquisition, suggesting the necessity of discouraging adolescents from relying on material possession strategies. Additionally, this research indicates materialistic values affect relationships with same-sex, but not opposite-sex peers, underscoring persistence of gender segregation characteristic in adolescent peer interactions (Leaper et al., 2022; Xie and Shi, 2009). It highlights the interpersonal challenges among interactions with same-sex peers that pose a prominent threat to adolescent life satisfaction.

Finally, this research contributes to the literature by highlighting gender similarity, rather than gender difference, in mechanisms for the association of materialistic values with life satisfaction. It is worthwhile to note that prior research presents inconsistent patterns regarding the moderation of gender on the mediating effect of peer relationship (e.g., Almquist et al., 2014; Luijten et al., 2023). This research demonstrates gender similarity in the mediating effect of same-sex peer relationship. Regarding the mediating effect of self-esteem, it is found similarly strong across both genders. This finding is different from our initial hypothesis, which expects a stronger mediating effect of self-esteem among girls than among boys. One possible interpretation for the failure of detecting a gender difference is the overall strong magnitude of the self-esteem effect across gender. Considering that this research is among the first few to examine the role of gender in mechanisms explaining the association of adolescent materialism with life satisfaction, these findings need to be replicated in the future.

## Practical applications of the findings

To our knowledge, the present study is among the first few to investigate the mechanisms underlying the path from materialism to adolescent life satisfaction. Findings have several practical applications. First, by enlightening educators and counselors on how materialism influences relational dynamics among adolescents, findings of this study can help design relationship and family counseling practices. The results indicate that same-sex, but not opposite-sex peer relationship in a young person's life, strongly mediates the association of materialistic values with his/her life satisfaction. To improve one's social relationship with same-sex peers and furthermore wellbeing, a piece of advice is to dematerialize. Moreover, given this finding, counselors and educators could address materialistic-related conflicts and harms by developing strategies to improve same-sex interpersonal relationships. Relationships with significant same-sex peers should also be assessed on an ongoing basis.

Second, this research demonstrates that, as compared to same-sex peer relationship, self-esteem is a more influential mediator explaining the connection between materialistic values and life satisfaction. This research brings awareness of the consequences of materialistic values endorsement on adolescent internalized self-evaluations. These findings implicate that educational programs or psychological interventions should focus on strengthening adolescent self-esteem as a means of mitigating the negative impacts of materialism on life satisfaction. Young individuals should be encouraged to engage in self-esteem-enhancing interventions such as self-affirmation practices (Fiska et al., 2024) and self-compassion techniques (Herrera and Turk, 2025), other than prioritizing the material possession importance as often promoted by marketing practitioners. Such efforts should help adolescents become more resilient toward the detrimental effects of materialism on self-esteem, thereby reducing its overall negative impacts on adolescent wellbeing.

Finally, this research helps raise awareness of the adverse effects of materialistic values endorsement and, thus, should provide guidance to marketers and media content generators in creating marketing materials. In particular, in order to acquire and maintain sustainable customers for the long term, creating materials that entertain or inform about the benefits of consumer products, but not implicitly endorsing materialism or distorting young individuals' perceptions, standards, and values of material possessions is suggested.

## Limitations and future directions

Several limitations are addressed. First, the present research does not provide a test of causality (Rohrer and Murayama, 2023), given the non-experimental design. It is possible that the effects are confounded by third variables that are not controlled for, such as personality traits (Górnik-Durose, 2020; Otero-López et al., 2023), environmental factors (Dávila and Casabayó, 2025), or the quality of interpersonal relations outside of peer relationship (Trzcińska et al., 2024). For example, materialists with a high level of neuroticism experienced diminished wellbeing in comparison to materialism with a low level of neuroticism (Górnik-Durose, 2020). This personality trait of neuroticism also exacerbates compulsive behavior (Otero-López et al., 2023), a behavior often found among materialists and detrimental upon subjective wellbeing. Experimental design with controlling for the confounding variables would be needed in the future.

Second, the present research used data from Mexican-origin adolescents living in the United States, raising the question of whether the findings generalizable to other ethnicity groups in the United States (Galinha et al., 2023). Although previous research suggests that materialism-related processes are often universal (e.g., Bradshaw et al., 2023; Dittmar et al., 2014), personal values are still found to predict life satisfaction differently across cultures (Galinha et al., 2023). Future research is needed to replicate findings with inclusion of youth from different ethnicity.

Third, same-sex, but not opposite-sex, peer relationship is found as a significant mediator. This result may be partly due to the developmental period (i.e., adolescence) when young individuals prefer hanging out with same-sex peers (Leaper et al., 2022). As the opposite-sex peer interactions become more intense and frequent over time (Syafiyah et al., 2025), the importance of opposite-sex peer relationship may also increase, In early adulthood (ages 19–25+), for example, the critical developmental task involves the formation of intimate relationships and commitments (Erikson, 1994; Koenig et al., 2025; Syafiyah et al., 2025), the effect of opposite-sex peer relationship would possibly become strong. Future research is needed with a focus on youth in a different developmental period.

## Conclusion

Extant research has insufficiently examined the underlying mechanisms driving the negative associations between adolescents' materialistic values (MV) and wellbeing. This study aims to address this gap. We hypothesize that higher MV endorsement would predict lower self-esteem, which, as a mediator, would further lead to lower life satisfaction. Same-sex and opposite-sex peer relationships would be alternative mediators as well. In addition, the hypothesized mediating effect of self-esteem would be stronger among girls than among boys.

Using longitudinal data from Mexican-origin adolescents, the present study contributes to the literature by validating the roles of self-esteem and same-sex peer relationship as significant mediators for the negative relationship between MV and life satisfaction. These findings are consistent with our hypotheses on the mediating effects of self-esteem and same-sex peer relationship. Notably, self-esteem emerges as a relatively stronger mediator than same-sex peer relationship. However, the findings fail to corroborate our hypotheses on the mediating effect of opposite-sex peer relationship, nor the hypothesized gender difference in the mediating effect of self-esteem.

## Data Availability

The datasets presented in this study can be found in online repositories. The names of the repository/repositories and accession number(s) can be found below: available on the Open Science Framework at https://osf.io/tbc27?view_only=820b87fa6685402587fcd82b730263dd.
